# Lumbar lordosis in prone position and prone hip extension test: comparison between subjects with and without low back pain

**DOI:** 10.1186/s12998-017-0139-x

**Published:** 2017-03-16

**Authors:** Amir Massoud Arab, Arash Haghighat, Zahra Amiri, Fariba Khosravi

**Affiliations:** 1 Department of Physical Therapy, University of Social Welfare and Rehabilitation Sciences, Evin, Koodakyar Ave, Tehran, 1985713831 Iran; 2 University of Social Welfare and Rehabilitation Sciences, Evin, Tehran, Iran

**Keywords:** Low back pain, Lumbar lordosis, Movement pattern, Prone hip extension, Flexible ruler

## Abstract

**Background:**

Prone hip extension (PHE) is a common and widely accepted test used for assessment of the lumbo-pelvic movement pattern. Considerable increased in lumbar lordosis during this test has been considered as impairment of movement patterns in lumbo-pelvic region. The purpose of this study was to investigate the change of lumbar lordosis in PHE test in subjects with and without low back pain (LBP).

**Method:**

A two-way mixed design with repeated measurements was used to investigate the lumbar lordosis changes during PHE in two groups of subjects with and without LBP. An equal number of subjects (*N* = 30) were allocated to each group. A standard flexible ruler was used to measure the size of lumbar lordosis in prone-relaxed position and PHE test in each group.

**Result:**

The result of two-way mixed-design analysis of variance revealed significant health status by position interaction effect for lumbar lordosis (*P* < 0.001). The main effect of test position on lumbar lordosis was statistically significant (*P* < 0.001). The lumbar lordosis was significantly greater in the PHE compared to prone-relaxed position in both subjects with and without LBP. The amount of difference in positions was statistically significant between two groups (*P* < 0.001) and greater change in lumbar lordosis was found in the healthy group compared to the subjects with LBP.

**Conclusions:**

Greater change in lumbar lordosis during this test may be due to more stiffness in lumbopelvic muscles in the individuals with LBP.

## Background

Low back pain (LBP) is a world-wide health problem and the most common and pricey musculoskeletal disorder in the today’s societies [1, 2]. The prevalence of LBP is estimated to be between 10 and 80% depending on the population [3, 4].

During the past decades some investigators proposed the regulation of the motor system and movement pattern in evaluation and management of LBP [5, 6]. A balanced motor system is obtained from coordinated activity of synergist and antagonist muscles. Normal functioning of the trunk depends not only on passive joint mobility, but also on normal muscular activity and central nervous system adjustment. Muscles produce and control the movement and stabilize the spine, protecting if from extreme load during functional activities [6, 7].

With regard to this point of view, repetitive movements and long-term incorrect postures and movements can change muscle tissue characteristics and can lead to muscle dysfunction, altered movement pattern, pain and finally movement disorders [5]. Hence, the main emphasis has been recently placed on assessment of the changed movement pattern in patients with musculoskeletal pain and disorders such as LBP and on the important of achieving normal pattern of the movement for the prevention and treatment of LBP [7–11].

Several studies have demonstrated that LBP is associated with muscle imbalance and changed activation pattern of the lumbo-pelvic muscles during different tasks [12–15].

There are some clinical tests that assess the altered movement pattern in subjects with LBP. Prone hip extension (PHE) which was originally developed by Janda is a common and widely accepted test for measuring the lumbo-pelvic movement pattern [9]. The importance of PHE is that the pattern of the movement during this test has been theorized to simulate those used during functional movement patterns such as gait [5, 10]. In this test, a patient lies prone and lifts his leg, while keeping the knee straight. It is assumed that alterations in this pattern can decrease the stability of lumbo-pelvic region during walking and insert abnormal stress on lumbar lordosis, resulting in LBP [16]. Good reliability has been reported for PHE in detecting deviation of lumbar spine from the midline [17].

Muscle imbalance and altered activation of the lumbo-pelvic muscles has been reported during PHE test in patients with chronic LBP. Coordination between muscles in the lumbo-pelvic region is thought to balance the position of the pelvis in normal posture and during the lower limb or trunk movement. It has been assumed that overactivity of the erector spinae muscles and inhibition or delayed activity of the gluteus maximus produced anterior tilt in the pelvic and increased lumbar lordosis especially in person with lower cross syndrome. Excessive anterior pelvic tilt, lumbar rotation, lumbar hyperextension, increased lumbar lordosis and knee flexion during the PHE has been considered as abnormal movement pattern during PHE [5].

The original intent of the PHE as taught by Janda was as a screen of lumbar stability in order to observe the timing, overactivity and delayed response of the lumbo-pelvic muscles and also the timing of the lumbar spine movement with concomitant anterior pelvic tilting during PHE test. However, with regard to the altered activity of the lumbo-pelvic muscles in subjects with chronic LBP, increased lumbar lordosis during PHE test might be expected in these subjects. Oh et al. stated that patients with LBP performing these exercises are often seen doing both hip and excessive lumbar spine movements, inducing unwanted anterior pelvic tilt and lumbar lordosis [18]. They believed that hip extension exercise may lead to a hyperlordotic lumbar angle and excessive pelvic tilt, because of instability in the lumbar and pelvis and imbalances in surrounding muscles, accordingly suggested lumbar stability exercises to prevent unwanted lumbar spine hyperlordosis and pelvic movement substitution [18]. Arab et al. found, although not statistically significant, greater change in lumbar lordosis during prone knee flexion test (another accepted clinical test for assessment of the lumbo-pelvic movement patterns) in subjects with LBP compared to those without LBP [19].

However, to our knowledge, no study has investigated the change in lumbar lordosis during PHE in patients with chronic LBP. The purpose of this study was to investigate the change in the size of lumbar lordosis during PHE in subjects with and without chronic LBP.

## Methods

### Subjects

A two-way mixed design was used to investigate the lumbar lordosis changes during PHE in two groups of men: men with chronic non-specific LBP (*N* = 30, average age: 33.6 (SD = 7.27); range: 22-47 years old, average height: 163.1 (SD = 8.25) cm, average weight: 59.5 (SD = 10.34) kg) and men with no history of LBP (*N* = 30, average age: 22.33 (SD = 1.93); range: 19-27 years old years old, average height: 177.42 (SD = 5.54) cm, average weight: 71.9 (SD = 8.98) kg).

The subject population in this study was a sample of convenience. The LBP patients were referred by physiotherapy clinics and orthopedic specialist. The patients were included if they had a history of non-specific LBP for more than six weeks’ duration before the study date, or had intermittent (on and off) LBP with at least three previous episodes each lasting more than one week, during the year before the study [20]. The control group were evaluated and found to have no complaint of any pain in their pelvis, low back, thoracic and lower extremities.

The exclusion criteria in both groups were history of dyspnea, history of hip pain, dislocation or fracture, history of lumbar spine surgeries, history of anterior knee ligament injury or rupture, history of anterior knee pain, recent episodes of ankle sprain, leg length difference of more than 1 cm, inability to perform active PHE without pain, history of lower extremity injury in the past 3 months, shortness of hip flexors, positive neurological symptoms and cardiopulmonary disorders. Each eligible subject was enrolled after signing an informed consent form approved by the human subjects committee at the University of Social Welfare and Rehabilitation Sciences. Ethical approval for this study was granted from the internal ethics committee at the University of Social Welfare and Rehabilitation Sciences.

### Procedures

Lumbar lordosis was measured in two conditions: prone relaxed position, and during PHE. The lordosis was measured in prone position before and after hip extension, respectively. The dominant leg was chosen for investigation.

### Measuring lumbar lordosis

A standard flexible ruler was used to measure the size of lumbar lordosis in prone position before and after PHE. For this purpose, the position of subject was prone lying on a treatment table with the arms along the sides. The spinous process of L1 and base of sacrum was located by palpation and marked with removable stickers. A standard flexible ruler was fitted in subject’s lumbar curve, over the lumbar spinous processes of L1 – S1. The curve of the flexible ruler, resembling the degree of subject’s lumbar curvature, was graphed on a paper, noting where the two reference points for L1 and S1 were located. The method explained by others [20–24] was used to quantify the size of lumbar lordosis (Ɵ). Two points on the curve, depicting L1 and S1, were connected by a line (L). A vertical line (H), representing the height of the lumbar curve, bisected line L. The length of each line was computed in millimeters, and the values were used in the following formula to calculate the degree of lumbar lordosis

$$ \uptheta = 4\ \left[\mathrm{Arctan}\ \left(2\mathrm{H}/\mathrm{L}\right)\right] $$

A very high correlation (*r* = 0.92) has been found between size of lumbar lordosis measured by a flexible ruler and from lumbar X-rays [20–24]. In the past, the reliability of flexible curve for measurement of lumbar lordosis has been established [20].

### Ethical approval

This research was reviewed and was approved by the Human Subject Committee at University of Social Welfare and Rehabilitation Sciences.

### Data analysis

Statistical analysis was performed using SPSS version 16.00. We tested the difference in lumbar lordosis between positions and groups by using two-way mixed-design ANOVA, accounting for position (prone-relaxed vs. PHE), health status (LBP vs. no LBP) and interaction of position and health status effects. Independent *t*-test was used to compare the amount of change in lumbar lordosis between positions (PHE minus prone-relaxed) across subjects with and without LBP. Statistical significant was attributed to P value less than 0.05.

## Results

The demographic data for the subjects is showed in Table 1. Statistical analysis showed no significant difference in subjects’ age (*P* = 0.15), height (*P* = 0.28), weight (*P* = 0.56) and BMI (*P* = 0.26) among the two groups.

**Table 1:** Demographic data of the subjects in each group (Mean ± SD)

Variables	With no LBP (*n* = 30)	With LBP (*n* = 30)
Age (years)	22.33 ± 1.93	33.6 ± 7.27
Weight (kg)	75.23 ± 22.91	59.5 ± 10.34
Height (cm)	177.00 ± 5.54	163.1 ± 8.25
BMI (kg/m^2^)	21.05 ± 2.26	22.31 ± 3.31

*BMI* Body Mass Index

**Fig. 1:**
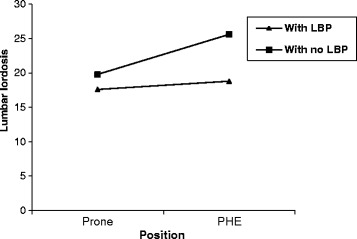
Lumbar lordosis in each position for two groups. *PHE* Prone hip extension, *LBP* Low back pain

Figure 1 depicts the average measurement scores for lumbar lordosis in each position for two groups. Detailed descriptive statistics (Mean SD) are presented in Table 2. The result of two-way mixed-design analysis of variance revealed significant health status by position interaction effect for lumbar lordosis at α = 0.05 (F = 27.41, *P* < 0.001). The main effect of test position on lumbar lordosis was statistically significant (F = 63.47, *P* < 0.001). Overall, the lumbar lordosis was significantly greater in the PHE compared to prone-relaxed position in both subjects with and without LBP (Table 2). The health status had significant effect of on lumbar lordosis (F = 25.30, *P* < 0.001). The mean difference in lumbar lordosis as measured by flexible curve between positions was 1.2 and 5.8 for subjects with LBP compared to those without LBP respectively. The amount of difference in positions was statistically significant between two groups (*P* < 0.001).

**Table 2:** The (Mean ± SD) scores of lumbar lordosis in each position for subjects with and without LBP

Variable	Group	Position		Difference between positions	*p*-value
		Prone	PHE		
Lumbar lordosis (degree)	With LBP (N = 30)	17.6 ± 2.07	18.8 ± 2.05	1.2	0.000
	With no LBP (*N* = 30)	19.8 ± 4.02	25.6 ± 5.9	5.8	0.000
*p*-value		0.23	0.000	0.000	

*PHE* Prone hip extension

*LBP* Low back pain

## Discussion

The current study compared the change in lumbar lordosis during PHE test between subjects with and without LBP. The results of this study indicate an increase in the size of lumbar lordosis during PHE compared to prone-relaxed position regardless of health status. However, greater change in lumbar lordosis was found in the healthy group compared to the subjects with LBP.

In this study, none of the subjects reported that pain was a limiting factor to perform PHE test, so, direct effects of pain can be minimized. However, nociception can influence the lumbar movement.

Lumbar extension and anterior rotation of the pelvis are often observed during hip extension motion. Increase in the size of lumbar lordosis during PHE found in both groups can be attributed to the accompanied lumbar extension during extension of hip. In theory, it is proposed that excessive anterior pelvic tilt, lumbar hyperextension and increased lumbar lordosis during the PHE are commonly seen as abnormal movement patterns in patients with chronic LBP. Investigators attributed these to muscle imbalance and changed activation of the lumbo-pelvic muscles [5]. They attributed excessive lumbar extension and hyperlordosis during PHE to deficit in controlling anterior pelvic rotation during hip extension because of muscular dysfunction in the lumbo-pelvic region [5, 18].

Sahrmann [5] proposed the concept of "relative flexibility or stiffness" that has been linked to uncontrolled movement, pain and pathology by causing direction related stress and strain during different functional movements in the patients with LBP. She suggested that increased stiffness of the anterior supporting structures of the thigh, hip and lumbar spine can result in compensatory exaggerated anterior pelvic tilt with lumbar extension motion during knee flexion or prone hip extension. In this study, stiffness in thigh and anterior supporting structures of the lumbar spine was not measured and just change in lumbar lordosis during PHE was measured.

Previous studies have demonstrated that patients with chronic or recurrent LBP use different strategies which are different from common one´s [25]. Scholtes et al. [25] found that during prone hip lateral rotation, subjects with LBP demonstrated a greater maximal lumbar-pelvic rotation angle compared to those without LBP, as the lumbar-pelvic region may move more frequently during the early ranges of lower limb movement in daily activities.

In this study, lumbar lordosis was significantly higher during PHE compared to prone relaxed position in subjects with or without LBP. However, we found that subjects with LBP, although not statistically significant, have a lower degree of lumbar lordosis change compared with those without LBP. The reason for this may be due to the increased hamstring stiffness and tightness in individuals with LBP. Previous studies have supported the change in mechanical behavior, extensibility and stiffness of hamstring muscles in subjects with LBP [20, 26–28]. Van Wingerden et al. considered hamstring muscle stiffness in patients with LBP as a compensatory mechanism to reduce pelvic instability and gluteal muscle weakness [29]. Investigators have attributed the increased activity of trunk muscles found in patients with LBP to functional adaptations following reduced lumbo-pelvic stability. Arab et al. [30] showed an increased EMG activity of the hamstring muscles during PHE in subjects with LBP compared to those without LBP. Because the hamstring muscle attaches to the ischial tuberosity, it is hypothesized that tightness and stiffness of these muscles may induce posterior pelvic tilt. However, in this study we did not measure the hamstring stiffness during PHE.

Some studies have found that patients with LBP have measurably greater stiffness than when they have no pain [31]. Others found that chronic LBP limits the maximal range of lumbar extension more than acute LBP [32]. The fact that change in lumbar lordosis during hip extension was smaller in patients with LBP compared to those without LBP may be due to the greater stiffness in subjects with chronic LBP.

Another area of concern in this topic may be fear-avoidance belief and pain avoidance in LBP. Fear may protect the individual from impending danger as it instigates defensive behavior. A large body of research revealed that LBP patients with pain-related fear report increased disability [33]. According to the fear-avoidance model of LBP, chronic LBP patients typically show submaximal performance and limited range of motion during physical activities such as straight leg raise, hip extension, trunk extension/flexion, and etc [11, 34, 35]. These findings suggest that performance during lumbo-pelvic extension, as measured by lumbar and hip excursions, may be influenced by individual differences in pain-related fear. Fritz and colleagues [35] have previously reported significant correlations between measures of fear avoidance behavior and some physical impairment that included lumbar range of motion (flexion, extension, average side bending, and average straight leg raise).

Lesser change in lumbar lordosis during PHE movement pattern in patients with LBP; found in this study, might be due to more limited hip and lumbar extension in these subjects as a results of fear-avoidance.

Some investigators proposed “guarding mechanism” during movement and activities in patients with LBP and stated that LBP patients show "guarded" movements during functional activities) [36]. This guarded movement during PHE may decrease lumbar lordosis changes in subjected with LBP [37].

### Limitations

We acknowledge some limitations. In this study the patients with chronic non-specific LBP were examined and other LBP patients (specific or acute LBP) were not examined. One other limitation of this study was this issue that LBP subjects were not categorized based on movement system impairment-based classified for LBP as described by Sahrmann [5]. It is suggested to investigate the lumbar lordosis change in LBP patients with different movement system impairment-based categories.

Another limitation of the study is that the original intent of the PHE as taught by Janda was as a screen of lumbar stability in order to observe the timing, overactivity and delayed response of the lumbo-pelvic muscles and also the timing of the lumbar spine movement with concomitant anterior pelvic tilting during PHE test. In this study we did not measure the electromyography (EMG) activity of the stabilizing and prime mover muscles during PHE to find the pattern of muscles recruitment. Another area of concern is that in this study, we did not measure lumbar -pelvic kinematics during PHE. Another issue is that the subjects with LBP were substantially older on average than those without LBP. This should be considered as the limitation in this study.

## Conclusion

This study investigated the change in lumbar lordosis during PHE test between subjects with and without LBP. The results of this study indicate an increase in the size of lumbar lordosis during PHE compared to prone-relaxed position in subjects with and without LBP. However, greater change in lumbar lordosis was found in the healthy group compared to the subjects with LBP.

## Abbreviations

LBP: Low back pain
PHE: Prone hip extension
